# Imported female genital schistosomiasis: a neglected health issue across borders

**DOI:** 10.7189/jogh.16.03002

**Published:** 2026-01-16

**Authors:** Kristin M Wall, Bellington Vwalika, William Evan Secor, Elisa García Vázquez

**Affiliations:** 1 Emory University, Department of Epidemiology, Atlanta, USA; 2 University of Zambia, School of Medicine, Lusaka, Zambia; 3 United States Centers for Disease Control and Prevention, Atlanta, USA; 4 Universidad de Murcia, Departamento de Medicina, Murcia, Spain

## Abstract

Female genital schistosomiasis (FGS) is one of the most neglected tropical diseases in the world, affecting over 56 million women and girls in Africa alone. It is a sequala of *Schistosoma haematobium* infection and is characterised by lesions on the cervix and other reproductive structures. Schistosomiasis and FGS are prevalent in women living in, migrating from, or traveling to *Schistosoma haematobium*-endemic countries. FGS is associated with significant morbidity, including adverse pregnancy outcomes. Unfortunately, imported schistosomiasis and FGS often remain undiagnosed or are diagnosed only at late stages of disease progression, months to years after arrival in non-endemic settings. This is due to limited diagnostic and screening test availability for schistosomiasis and an absence of awareness and guidelines to diagnose imported FGS, especially among sexual and reproductive health providers. Fragmented care pathways between infectious disease, travel/tropical medicine, and reproductive health services further contribute to missed diagnoses, while structural and social inequities due to migration status and stigma lead to barriers in FGS diagnosis and management.

Female genital schistosomiasis (FGS) is one of the most neglected tropical diseases (NTDs) in the world, affecting more than 56 million women and girls in Africa alone, where 90% of cases occur [1]. It is characterised by lesions on the cervix and other reproductive structures that develop after infection with *Schistosoma haematobium*, a parasitic trematode flatworm transmitted through contact with infectious freshwater. Characteristic FGS lesions form as an inflammatory response to parasitic eggs trapped in body tissue and can persist even after the schistosome infection has been treated and resolved. Though active *S. haematobium* infection is relatively straightforward to diagnose *via* urine filtration to identify parasitic eggs, the clinical diagnosis of FGS is more difficult, requiring colposcopic examination of the cervix and vaginal walls by a trained expert for identification of characteristic lesions [2]. The condition is associated with significant morbidity including infertility [3], ectopic pregnancy and other adverse pregnancy outcomes [4–6], and HIV risk [7–9].

In Europe, imported schistosome infections may occur in migrants from, and travelers to, countries where schistosomiasis is endemic. The European Network for Tropical Medicine and Travel Health estimated that over half of imported schistosomiasis cases occur among non-European immigrants, 33% among European travellers, and 16% among long-term expatriates [10]. Recent screening studies reported active schistosome infections in 10–12% of adults [11] and children [12] migrating to Spain, and 17–21% of adults migrating to Italy [13,14]. Among migrants into Italy presenting to care with signs and symptoms of infection, 47% were diagnosed with schistosomiasis [15].

Though data on imported FGS prevalence are more limited, a 2025 empirical treatment study in Spain found that chronic schistosomiasis is common among African migrants, who had been in Europe roughly seven years [16]. A prospective population-based study of long-staying female African migrants in Europe found very high prevalence of FGS-related symptoms, including dysmenorrhea (79%), leukorrhea (74%), and amenorrhea (47%) [17].

Unfortunately, a common theme across studies describing imported schistosomiasis and FGS is underdiagnosis or late-stage diagnosis. A multicentre study in Italy found a median time from migrant arrival to diagnosis of 9.2 years for asymptomatic schistosomiasis and 1.3 years for symptomatic patients [18]. A literature review of FGS in travellers returning from *S. haematobium* endemic countries found correct diagnoses only occurred months to years after their return [19].

These delays are consequential. Early diagnosis and treatment are crucial for avoiding the development of potentially irreversible complications [20]. Treatment for both schistosomiasis and FGS is a single dose of 40 mg/kg praziquantel [21]. A meta-analysis of clinical trials in endemic areas showed this regimen has a cure rate of 77% for *S. haematobium* [22]; in non-endemic areas where reinfection is less likely, cure rates may be higher. However, questions remain about the optimal treatment regimen for imported schistosomiasis [23] and whether this treatment regimen is effective for FGS [24]. Data from Zambia showed that FGS lesion severity decreased in 60% of women nine months post-treatment and completely resolved in 23%; those with greater disease severity at treatment were less likely to resolve lesions, underscoring the need for early detection [25].

There are several reasons for delayed diagnosis or underdiagnosis of imported schistosomiasis and FGS, including institutional and service delivery barriers, and structural and social inequities (**Table 1**).

**Table 1:** Summary of key challenges perpetuating non-diagnosis or diagnostic delays in imported schistosomiasis and FGS and recommendations

Specific challenges	Recommendations
Institutional and service delivery barriers	
*Absence of screening, diagnostic, and management guidelines for FGS*	Develop case definitions and guidelines for the routine surveillance, diagnosis, treatment, and management of imported FGS
*Provider awareness gaps and curricula omissions*	Develop culturally competent training curricula for sexual and reproductive health professionals, including visual identification of FGS
*Limited screening and diagnostic tool availability*	Ensure availability of diagnostic tests (urine filtration, serology) for schistosomiasis
	Adapt screening tools based on travel history, signs, and symptoms to improve disease identification in clinical practice
*Fragmented care pathways*	Strengthen cross-referral systems across infectious disease, gynaecology, and sexual and reproductive health
Structural and social inequities	
*Barriers due to migrant status*	Policy changes supportive of meeting migrant women’s health needs, including translation services and financial assistance programmes
*Female stigma and misdiagnosis*	Ensure provider trainings are culturally sensitive and sex-specific

FGS – female genital schistosomiasis

## INSTITUTIONAL AND SERVICE DELIVERY BARRIERS

While recent consensus statements and guidelines describe screening, diagnosis, and management for imported schistosomiasis [26–29], case definitions and guidelines for the routine surveillance, screening, diagnosis, treatment, and management of imported FGS have not yet been developed. This limits detection and disease management, as well the availability of epidemiologic data on FGS prevalence and impact. By contrast, other NTDs such as imported chikungunya and dengue are covered by more advanced surveillance systems in Europe [29], in part due to their higher potential for domestic transmission [30]. However, though the region is generally considered non-endemic for schistosomiasis [31], local transmission has been documented in Corsica, demonstrating that parts of Europe have conditions conducive to maintaining the parasite’s life cycle [32].

Health providers’ awareness of schistosomiasis and FGS in non-endemic countries is also low. European medical curricula rarely cover NTDs outside of tropical and travel medicine specialties. As a result, schistosomiasis knowledge resides primarily in travel medicine or infectious disease clinics, while women with common FGS symptoms (discharge, bleeding, infertility, pelvic pain) are usually seen in sexual health or gynaecology settings. A survey of European healthcare workers showed higher knowledge of FGS among health providers with infectious disease and travel/tropical medical training (69%) than among gynaecology specialists (only 40%) [33]. A review of FGS case reports in returned travellers found no cases in which clinicians consider FGS in differential diagnoses of genital or urinary symptoms, and that diagnoses were made long after travel exposure [19]. By contrast, mosquito-borne NTDs fit more neatly into vector-borne infectious disease training frameworks and do not require cross-specialty training.

The availability of diagnostic tests and screening tools for schistosomiasis (*e.g.* urine filtration for parasitic eggs, serology for infection history) is also often limited. An assessment of nine Italian infectious and tropical diseases centres found that, despite schistosomiasis being one of the most commonly diagnosed NTDs, serological tests were unavailable at two facilities, while praziquantel treatment was not consistently available at three facilities [34]. Additionally, while screening algorithms comprised of exposure risk factors and signs/symptoms have shown promise for FGS screening [35] and may be effectively used in combination with serologic screening tests, such screening tools have not been coupled with travel history, signs, and symptoms for identification of imported FGS.

## STRUCTURAL AND SOCIAL INEQUITIES

Migrant women face intersecting barriers to healthcare due to structural and social inequities related to migration status, sex, and race. Recent systematic and scoping reviews of healthcare barriers faced by migrant women highlight that they, on average, have lower socioeconomic status, including lower health insurance coverage than residents, face higher financial barriers, and require language translation services that are often unavailable in healthcare settings [36,37]. Legal precarity, fear of deportation, discrimination, and medical mistrust are also frequently reported barriers to routine healthcare [37]. These reviews noted that translation services, increasing health provider cultural competency, addressing economic barriers through assistance programmes, and establishment of inclusive policies to ensure non-discrimination and confidentiality among migrant populations could help mitigate these issues.

Finally, the stigma associated with seeking care for gynaecological symptoms may delay treatment [38–40]. While many other common imported NTDs affect both sexes and do not cause reproductive health symptoms, common FGS symptoms, including vaginal discharge, genital itching, abdominal and pelvic pain, and pain during urination, may be confused with sexually transmitted infections which are often stigmatised, especially in women [38–40]. African women have reported that other FGS symptoms including pain during sexual intercourse, sub-fertility, and incontinence can cause social stigma leading to isolation, poor mental health, and delayed healthcare seeking [38–40].

## RECOMMENDATIONS

Imported schistosomiasis and FGS are severely neglected diseases with substantial clinical burdens, deserving of more attention in endemic regions and non-endemic regions with large populations of African migrant women. There is an urgent need to develop case definitions and guidelines for the routine surveillance, diagnosis, treatment, and management of imported FGS. This would support not only improved disease identification and management, but could also generate important data for advocacy and policy recommendations. Additionally, culturally- and female-sensitive training curricula are needed for infectious disease and sexual and reproductive health professionals, including training in visual identification of FGS lesions. Availability of urine filtration and serology (including in sexual and reproductive health settings), adapting screening questionnaires, and strengthening referral systems across infectious disease, travel/tropical medicine, and sexual and reproductive health services would support routine service delivery. Finally, policy changes supportive of meeting migrant health needs in accordance with the World Health Organization’s evidence-based recommendations [41], including non-discrimination policies, translation services, and financial assistance programmes would support migrant access to healthcare.
